# Temperature-Responsive Gelation of Type I Collagen Solutions Involving Fibril Formation and Genipin Crosslinking as a Potential Injectable Hydrogel

**DOI:** 10.1155/2013/620765

**Published:** 2013-10-05

**Authors:** Shunji Yunoki, Yoshimi Ohyabu, Hirosuke Hatayama

**Affiliations:** Biotechnology Group, Tokyo Metropolitan Industrial Technology Research Institute, 2-4-10 Aomi, Koto-ku, Tokyo 135-0064, Japan

## Abstract

We investigated the temperature-responsive gelation of collagen/genipin solutions using pepsin-solubilized collagen (PSC) and acid-solubilized collagen (ASC) as substrates. Gelation occurred in the PSC/genipin solutions at genipin concentrations 0–2 mM under moderate change in temperature from 25 to 37°C. The PSC/genipin solutions exhibited fluidity at room temperature for at least 30 min, whereas the ASC/genipin solutions rapidly reached gel points. In specific cases PSC would be preferred over ASC as an injectable gel system. The temperature-responsive gelation of PSC/genipin solutions was due to temperature responses to genipin crosslinking and collagen fibril formation. The elastic modulus of the 0.5% PSC/genipin gel system could be adjusted in a range of 2.5 to 50 kPa by the PSC and genipin concentrations, suggesting that a PSC/genipin solution is a potential injectable gel system for drug and cell carriers, with mechanical properties matching those of living tissues.

## 1. Introduction

Injectable hydrogels (in situ formed hydrogels) are promising substrates for tissue-engineering applications because of their ability to provide biodegradable scaffolds with physical properties that match those of living tissues and to control drug delivery with minimally invasive operations [1]. Thermo- and pH-responsive polymers have been studied extensively with the aim of achieving physical crosslinking that responds to physiological conditions (pH, neutral; temperature, 37°C) without using cytotoxic stimulation [2]. Such physical crosslinking poses little or no cytotoxicity [3–5]. In contrast, chemical crosslinking employs free radicals, Michael-type addition, or Schiff-base reactions [1]. A physically crosslinked injectable hydrogel is thus considered to be more biocompatible.

Type I collagen (abbreviated here as “collagen”) has been used as a physically crosslinked injectable hydrogel. Collagen molecules are stable in acidic solutions at low temperatures and are capable of forming nanofibrils that respond to body temperature and neutral pH [6, 7], permitting use of collagen as an injectable hydrogel [8, 9]. Among biocompatible polymers, collagen is one of the most preferred for gel systems because of its many advantages, including biocompatibility, bioabsorbability, and cell adhesiveness [10]. Injectable collagen hydrogels have been successfully used for soft-tissue augmentation [11, 12], drug delivery carriers [13, 14], and hard-tissue augmentation [15]. Collagen offers an ideal injectable gel system from the biological perspective, but the weak mechanical properties of collagen gels limit their practical applications. The complex modulus of cell-encapsulated collagen gel is less than 20 Pa at a collagen concentration of 0.2% [16], much lower than that of living tissues. The shear storage modulus of acellular collagen gel is no more than 150 Pa [17]. However, conventional chemical crosslinking cannot be used for injectable collagen gel systems because the cytotoxic crosslinkers are directly exposed to living cells and tissues.

Regarding the potential cytotoxicity of chemical crosslinkers, natural crosslinkers such as enzymes (e.g., transglutaminase [18], lysyl oxidase [19]) and plant extracts (e.g., genipin [20–26]) have been expected to provide alternatives and reduce cytotoxicity of biomaterials. Among them, genipin shows rapid crosslinking of collagen comparable to glutaraldehyde [21]. Genipin is a natural crosslinker of proteins extracted from gardenia fruit, and its cytotoxicity has been reported to be much lower than that of most commonly used synthetic crosslinkers.

Mechanical characterization of collagen fibrillar gels crosslinked by genipin has been firstly reported by Sundararaghavan et al. [24]. Macaya and coworkers [25] have recently reported an injectable collagen gel system using genipin as a crosslinker and acid-solubilized collagen (ASC) as a substrate. The mechanical properties of the gels (of storage modulus approximately 250 Pa) were much better than those of collagen fibrillar gels without chemical crosslinking [16].

However, the fluidity of the collagen/genipin solution at room temperature was unknown. We expected that the slow fibril formation of pepsin-solubilized collagen (PSC) compared with ASC [27, 28] would contribute to maintain fluidity at room temperature. PSC is prepared from ASC or collagenous tissues by pepsin digestion and lacks nonhelical terminal regions (telopeptides) while triple-helical conformation is intact [29]. Conventional biomaterial processing has employed PSC rather than ASC because of its processing-related [29] and immunological benefits [30]. The temperature responsiveness of genipin crosslinking is as important for gelatin properties as that of collagen fibril formation. A previous study [21] showed that genipin crosslinking of collagen was similar in 72 h reaction at temperatures of 25–45°C; however, the temperature responsiveness on second-minute timescale is still unknown. Sol to gel transition of injectable collagen (i.e., fibril formation) occurs on such timescale.

In the present study, we investigated the fluidity of a collagen/genipin solution at room temperature and its gelation response to physiological temperatures using pepsin-solubilized collagen (PSC) as a substrate. We hypothesized that an injectable collagen gel system using PSC confers sufficient and prolonged fluidity at room temperature. We also investigated the temperature response of genipin crosslinking. The potential of PSC/genipin solution as an injectable gel system is discussed.

## 2. Materials and Methods

### 2.1. Materials

Materials comprise PSC from porcine skin in dilute HCl (1% solution; Nipponham, Japan), ASC from porcine tendon in dilute HCl (0.3% solution; Nitta Gelatin, Japan), chitosan powder (Chitosan 500; Wako Pure Chemical Industries, Japan), genipin (Wako Pure Chemical Industries, Japan), glutaraldehyde (25% solution; Wako Pure Chemical Industries, Japan), and phosphate-buffered saline (PBS) tablets (Sigma-Aldrich, MO, USA).

### 2.2. Preparation of Solutions

The PSC (1%) and ASC (0.3%) and solutions were used with no pretreatment. According to the manufactures' data by electrophoresis, PSC and ASC contained 11 wt% and a trace amount of type III collagen, respectively. PSC solutions of 0.3 and 0.6% were prepared by dilution of the 1% PSC solution with pH 3 dilute HCl. A 1% chitosan solution was prepared by dissolution of chitosan powder in 100 mM acetic acid solution. Genipin solutions at concentrations ranging from 1 to 10 mM were prepared by dissolution of genipin powder in 2 × PBS and held for 2 weeks in the refrigerator prior to use for the following examinations. GA solutions at a concentration of 12 mM were prepared by dilution of 25% GA solution with 2 × PBS.

### 2.3. Dynamic Viscoelastic Tests

#### 2.3.1. Apparatus

Changes in fluidity and gelation processes were monitored by dynamic viscoelastic tests with a rheometer (HAAKE MARS III; Thermo Fisher Scientific Inc., MA, USA). The temperature was sensitively controlled by a Peltier controlled plate. Two types of sensors were used: a double-cone sensor DC60/1Ti (diameter of 60 mm, cone angle of 1°) and a parallel-plate sensor HPP35S (diameter of 35 mm, with surface serrated to avoid slippage of gels during oscillation). The double-cone sensor has a shear surface twice as high as that of a conventional cone plate, stabilizing viscoelastic data of low viscous samples by enhanced sensitivity. A lid was used to minimize drying of the solutions during measurement at room temperature, enabling precise viscoelastic measurements for more than 30 min. 

#### 2.3.2. Sample Preparations

PSC/genipin and ASC/genipin solutions were prepared as follows. PSC and ASC solutions (3 g) at concentrations of 0.3, 0.6, or 1% were placed in 15 mL polypropylene tubes and maintained at 4°C. Three milliliters of precooled genipin solution or GA solution (concentrations of 0–10 mM) at 4°C was added to the collagen solution and vortexed immediately to prepare neutral collagen solution containing genipin or GA in 1 × PBS. The pH of the mixtures of collagen/genipin and collagen/GA mixtures was 7.2; there was a slight decrease from the pH of PBS (7.4). We therefore employed the simple gelation protocol on the assumption that such a slight decrease in pH hardly affected collagen fibril formation and chemical crosslinking. We think that PBS is appropriate solvent for in vitro experiments of injectable collagen because NaCl concentration has a strong effect on collagen fibril formation. The NaCl concentration in PBS (140 mM) is equal to those of saline and conventional cell culture media as possible solvents of injectable collagen. A small aliquot of the collagen/genipin solution at collagen concentrations of 0.15, 0.3, or 0.5% was placed on the rheometer.

 As for the chitosan/genipin solution, 3 g of the acidic chitosan solution at a concentration of 1% was placed in a 15 mL polypropylene tube and kept at 4°C. Following mixing with genipin solution or GA solution according to the procedure described above, 0.5% chitosan solutions containing genipin or GA were obtained.

#### 2.3.3. Test Conditions

Dynamic viscoelastic tests were initiated in 60 s after the collagen and genipin solutions were mixed, using the following test conditions.

(*1) Fluidity Test.* Oscillation at a frequency of 1 Hz was applied at a constant shear stress of 0.1 Pa and constant temperature of 25°C using the double-cone sensor. The condition was in the linear viscoelastic regime of the collagen/genipin solutions. Changes in storage modulus (*G*′) and loss modulus (*G*′′) were registered. The gel point was determined as the point at which *G*′ and *G*′′ intersected in their time-course change. Fluidity of solutions was defined as *G*′ < *G*′′.

*(2) Temperature-Responsive Gelation Test.* Oscillation at a frequency of 1 Hz was applied at a constant shear deformation of 0.01 using the parallel-plate sensor. The temperature was kept at 25°C for intervals ranging from 10 to 40 min, increased from 25 to 37°C over 30 s, and then kept at 37°C for 60 min. Changes in *G*′ and *G*′′ were recorded. The oscillation conditions were designed to produce a linear viscoelastic regime in the collagen/genipin solutions after the gel points.

### 2.4. Penetration Test

#### 2.4.1. Apparatus

Penetration tests of the PSC/genipin gels were conducted with a mechanical tester (TA.XTplus; Stable Micro Systems, UK). A cylindrical stainless probe (5 mm in diameter) was used.

#### 2.4.2. Sample Preparation

PSC solutions of 0.15% and 0.5% containing various concentrations of genipin were prepared according to the sample preparation for the dynamic viscoelastic tests. The solutions were degassed by centrifugation for 30 s after vortexing, and aliquots (4 mL) were poured into petri dishes (diameter 55 mm) and placed on the surface of a water bath at a temperature of 37°C. After 30 min of warming, the dishes were sealed with a paraffin film to avoid drying of the gels, moved to an incubator at a temperature of 37°C, and incubated for 72 h to complete gelation. 

#### 2.4.3. Penetration of PSC/Genipin Gels

Stiffness of the matured PSC/genipin gels was determined by a penetration test using the mechanical tester. The centers of the gels (*n* = 5) in the biological plates were probed with the cylindrical probe at a cross-head speed of 0.2 mm/s, and stress-strain curves were obtained. The sensitivity of the mechanical tester was set at 10 Pa. The elastic modulus was calculated from the slope of the stress-strain curve in its linear region (strain from 0.005 to 0.04), in which consolidation of the gels by the biological plates did not affect the mechanical data.

### 2.5. Scanning Electron Microscopy (SEM) Observation

The morphologies of collagen fibrils in the gels were observed by SEM. Fixation and drying of the gels were performed following our previous report using glutaraldehyde and *t*-butyl alcohol [31]. In brief, the gels prepared, as described in Section 2.4.2, were fixed with 2.5% glutaraldehyde solution and subsequently dehydrated by sequential immersion in 20, 50, 75, 90, 95, and 99% ethanol. The immersion in 99% ethanol was repeated three times. The ethanol in the dehydrated gel was thoroughly substituted with *t*-butanol and subjected to drying in vacuo. The interiors of the dried gels were exposed by peeling with tweezers and coated with Au. SEM measurements were performed with a Miniscope TM3000 (Hitachi Ltd., Tokyo, Japan) at an acceleration voltage of 5 kV.

## 3. Results

### 3.1. Fluidity Tests of PSC/Genipin Solutions at Room Temperature

**Figure 1:**
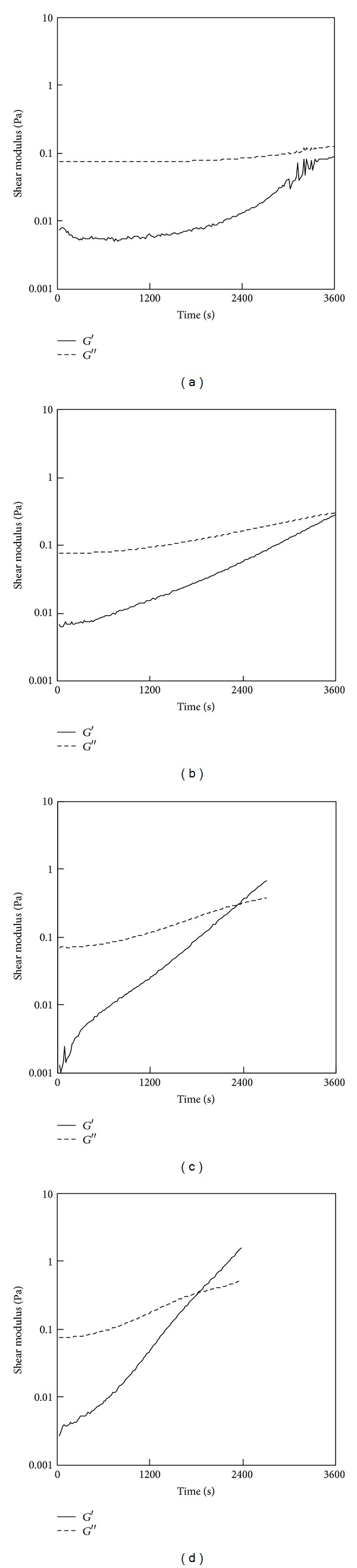
Results of a fluidity test for the 0.15% PSC/genipin solutions containing various concentrations of genipin: (a) 0 mM, (b) 0.5 mM, (c) 1 mM, or (d) 2 mM. Temperatures were maintained at 25°C.

**Figure 2:**
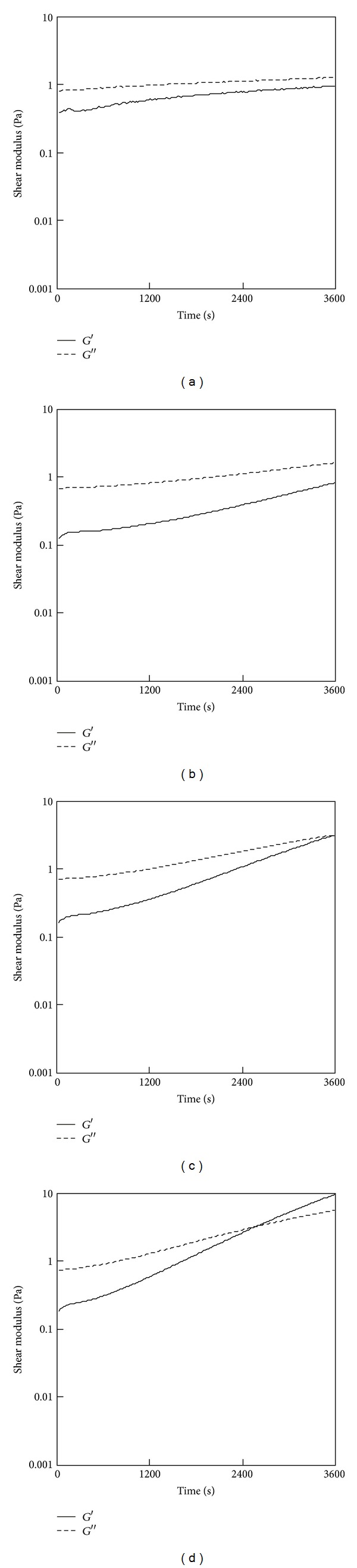
Results of a fluidity test for the 0.5% PSC/genipin solutions containing various concentrations of genipin: (a) 0 mM, (b) 0.5 mM, (c) 1 mM, or (d) 2 mM. Temperatures were maintained at 25°C.

Figure 1 shows the results of the fluidity test for the 0.15% PSC/genipin solutions containing various concentrations of genipin (0–2 mM). In the absence of genipin, *G*′′ was greater than *G*′ over the entire range of timepoints investigated (Figure 1(a)), exhibiting a viscous nature. Turbidity was not observed, providing evidence that fibril formation of PSC did not occur in 60 min at 25°C. *G*′ and *G*′′ showed time-dependent increases in the presence of genipin, where the gel point, defined as the time at which *G*′ and *G*′′ were equal, became shorter with the increasing concentration of genipin (Figures 1(a)–1(d)). The reduced gel points indicated that genipin gradually crosslinked collagen monomers. The gel point was determined to be 2190 ± 180 s (mean ± SD; *n* = 3) at a genipin concentration of 1 mM. The gel points of the 0.5% PSC/genipin solutions were longer than those of the 0.15% PSC/genipin solutions at the same genipin concentrations (Figure 2). The gel point at a genipin concentration of 2 mM was determined to be 2490 ± 270 s (mean ± SD; *n* = 3). At a genipin concentration of 1 mM, the solution did not reach a gel point within 60 min in triplicate experiments. 

### 3.2. Fluidity Tests of ASC/Genipin Solutions at Room Temperature

**Figure 3:**
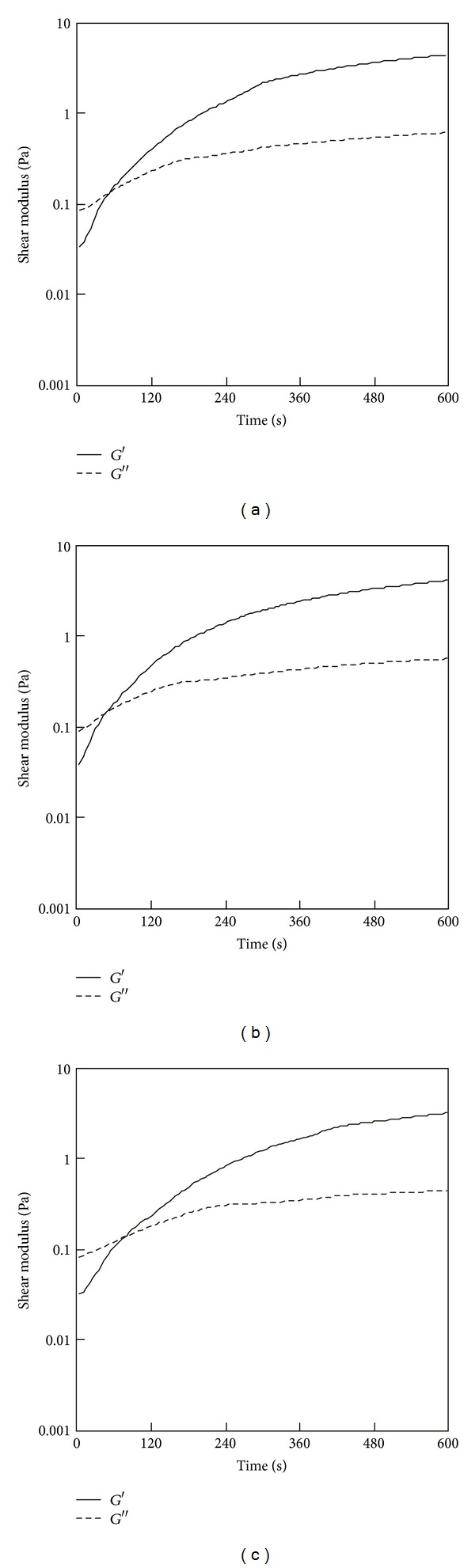
Results of a fluidity test for the 0.5% ASC/genipin solutions containing various concentrations of genipin: (a) 0 mM, (b) 0.5 mM, (c) 1 mM, or (d) 2 mM. Temperatures were maintained at 25°C.

Fluidity tests were also conducted for 0.15% ASC/genipin solutions (Figure 3). The solution became turbid immediately after the ASC solution was mixed with the genipin solution. The immediate increase in *G*′′ resulted in the gel points within 60 s (Figures 3(b) and 3(c)). Even when the ASC solution was mixed with PBS containing no genipin, the solution reached a gel point within 60 s (Figure 3(a)). These results indicated that the immediate increases in *G*′ of 0.15% ASC/genipin solutions were predominantly because of active fibril formation of ASC at neutral pH.

### 3.3. Temperature-Responsive Gelation of PSC/Genipin Solutions

**Figure 4:**
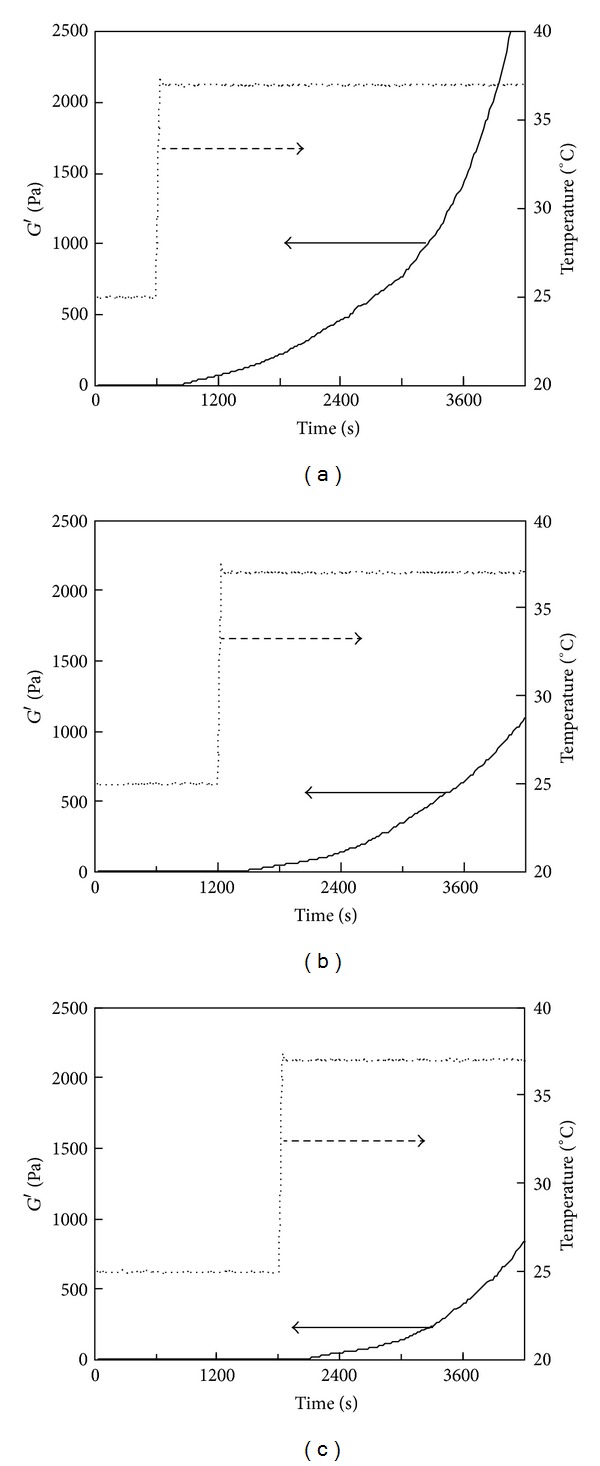
Storage modulus (*G*′) of the 0.5% PSC/1 mM genipin solution measured by a temperature-responsive gelation test. The temperature was maintained at 25°C for (a) 600, (b) 1200, and (c) 1800 s and then increased to 37°C.

**Figure 5:**
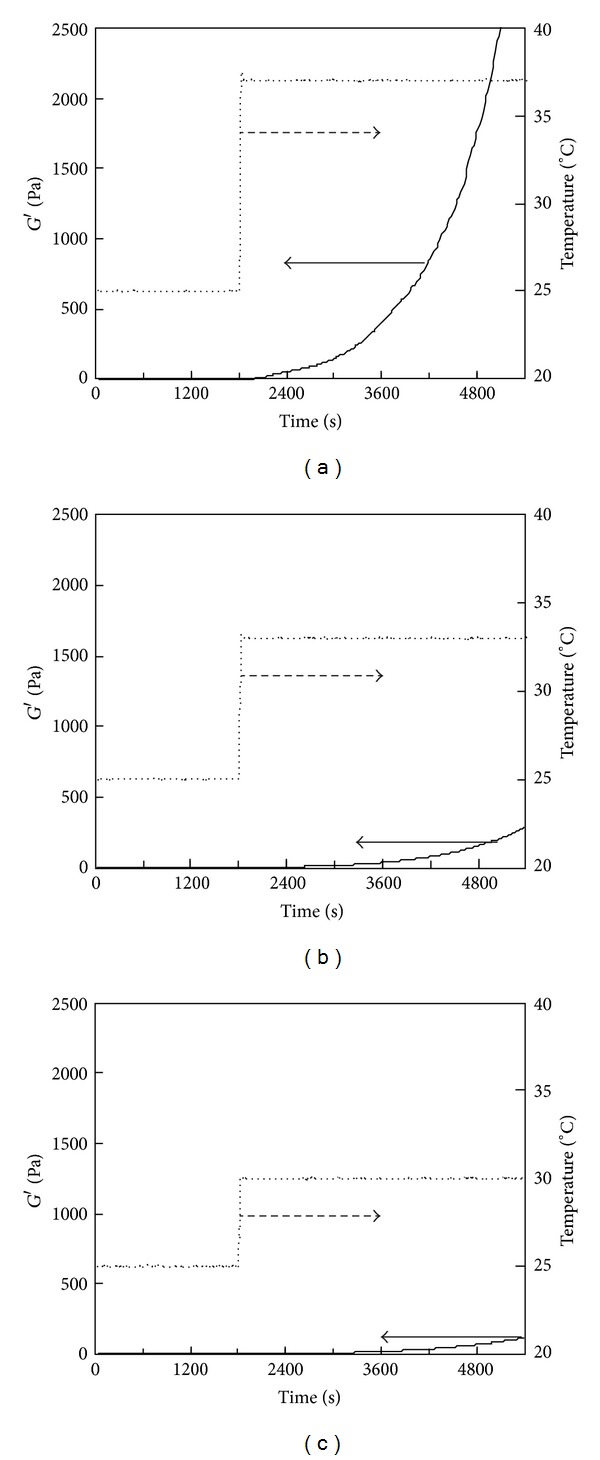
Storage modulus (*G*′) of the 0.5% PSC/1 mM genipin solution measured by a temperature-responsive gelation test. The temperature was maintained at 25°C for 1800 s and then increased to (a) 37, (b) 33, or (c) 30°C.

Temperature-responsive gelation tests were conducted for the 0.5% PSC/1 mM genipin solutions. The intervals at 25°C were set at 600, 1200, and 1800 s, during which the solutions remained viscous (Figure 2(c)). Sharp increases in *G*′ were observed approximately 100 s after the solutions reached the target temperature (37°C) (Figure 4), indicating that gelation was triggered by the increase in temperature to physiological temperature. The rates of modulus increase were not affected by the length of intervals at 25°C. Lower target temperatures (33 and 30°C) also were investigated.  Figure 5 shows the results of temperature-response tests employing target temperatures of 30, 33, and 37°C and an interval of 1800 s. The rate of increase in *G*′ decreased with the target temperature, supporting the hypothesis that this gel system is temperature-responsive. The turbidity of the gels, corresponding to the actual fibril formation rate, tended to decrease with increase in the concentration of genipin.

### 3.4. SEM Observation

**Figure 6:**
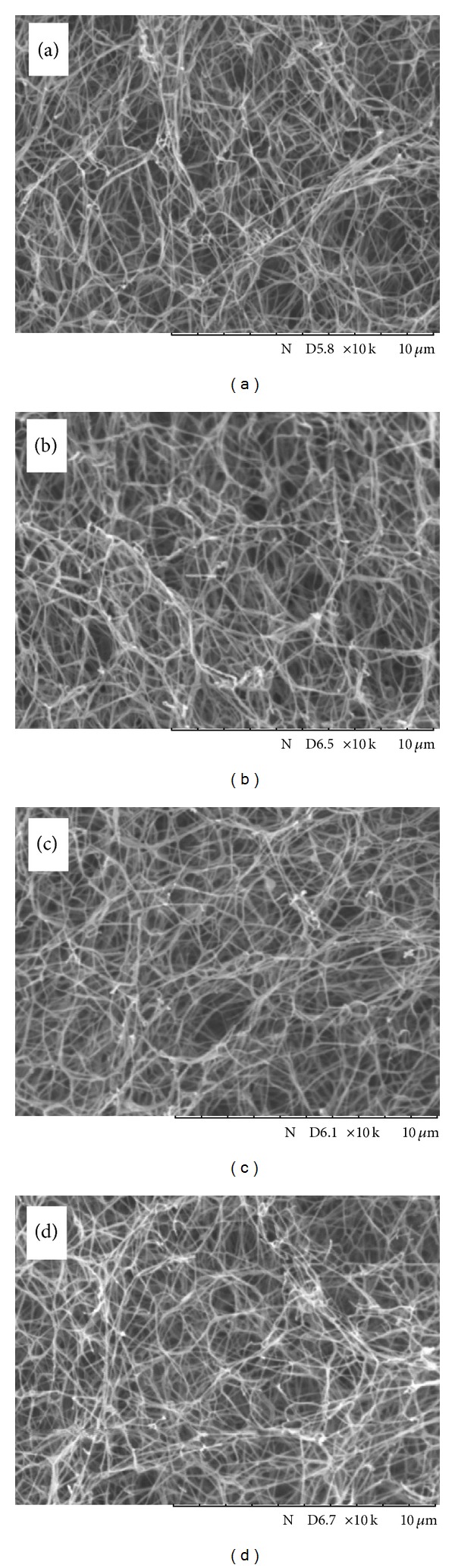
SEM images of cross-sections of dried PSC/genipin gels at genipin concentrations of (a) 0 mM, (b) 0.5 mM, (c) 1 mM, and (d) 2 mM.

Figure 6 shows SEM images of the cross-sections of dried PSC/genipin gels. A drying process using *t*-butyl alcohol sublimation enabled observation of collagen fibrils in the gels with minimal destruction. PSC/genipin gels at genipin concentrations of 0.5, 1, and 2 mM (Figures 6(b)–6(d)) showed networks of collagen nanofibrils resembling those obtained from PSC gel containing no genipin (Figure 6(a)). We must note that the apparent fibril density in the SEM images became similar by the alcohol dehydration and sublimation process.

### 3.5. Effects of Collagen and Genipin Concentrations on Temperature-Responsive Gelation

**Figure 7:**
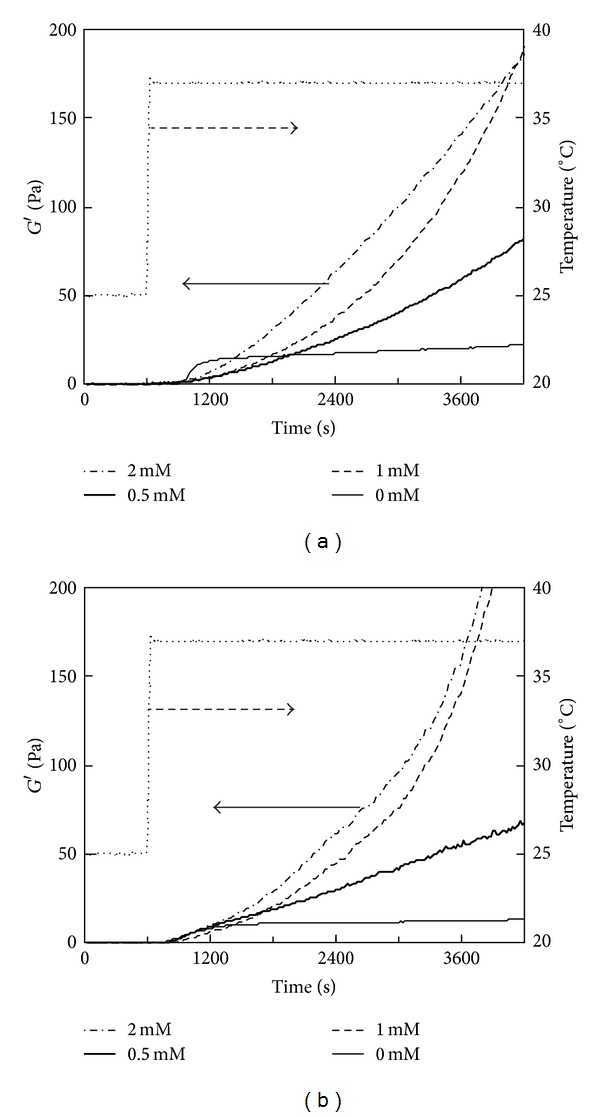
Storage modulus (*G*′) of the 0.5% PSC/genipin solution measured by a temperature-responsive gelation test. The temperature was maintained at 25°C for 600 s and then increased to 37°C. Numbers in figures indicate genipin concentrations.

**Figure 8:**
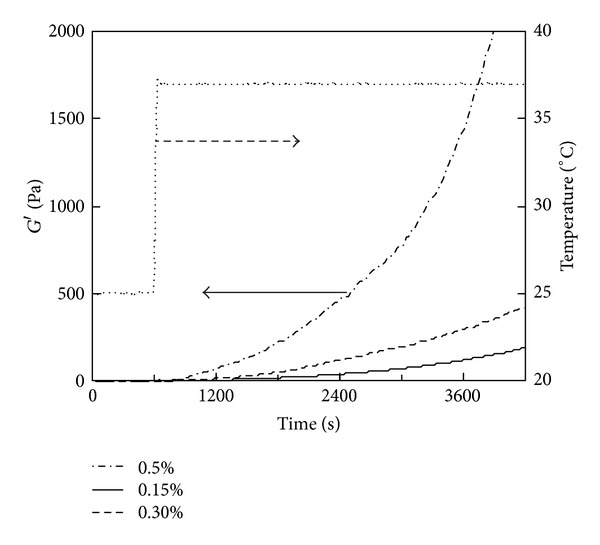
Storage modulus (*G*′) of PSC/1 mM genipin solutions measured by a temperature-responsive gelation test. PSC concentrations were (a) 0.15% and (b) 0.5%. The temperature was maintained at 25°C for 600 s and then increased to 37°C. Numbers in figures indicate PSC concentrations.

The effect of the genipin concentration on gelation was investigated by temperature-responsive gelation tests for 0.5% and 0.15% PSC solutions. No significant increase in *G*′ was observed in the intervals at 25°C for all the solutions (Figure 7). The gel points were almost independent of genipin concentrations: all the 0.15% PSC solutions containing 0–2 mM of genipin passed through gel points in the increasing temperature from 25°C to 37°C (Figure 7(a)). As for the 0.5% PSC solutions containing genipin, gel points were achieved in 160–180 s after the temperatures reached 37°C (Figure 7(b)). The rate of increase in *G*′ was proportional to the increase in the genipin concentration (Figure 7). The genipin concentration dependence was similar in 0.15% and 0.5% PSC solutions, although *G*′ values of 0.5% PSC gels were much higher than those of 0.15% gels. The effect of the collagen concentration on gelation was also investigated. The rate of increase in *G*′ accelerated as the PSC concentration increased from 0.15 to 0.5% (Figure 8).

### 3.6. Temperature Responsiveness of Genipin Crosslinking and Collagen Fibril Formation

**Figure 9:**
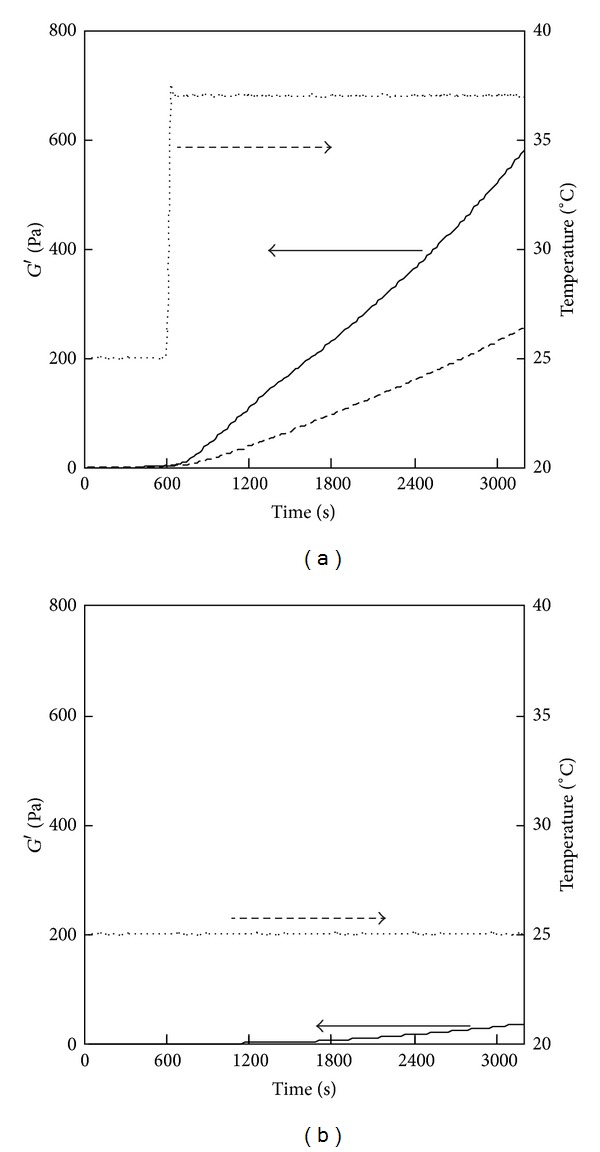
(a) Storage modulus (*G*′) of chitosan solutions measured by a temperature-responsive gelation test. Solid line, 0.5% chitosan/5 mM genipin solution; dotted line, 0.5% chitosan/6 mM GA solution. (b) Storage modulus (*G*′) of the 0.5% chitosan/5 mM genipin solution measured by a fluidity test at a constant temperature of 25°C.

To evaluate the temperature dependence of genipin crosslinking without the influence of collagen fibril formation, a 0.5% chitosan/5 mM genipin solution was subjected to the temperature-responsive gelation test. Chitosan is a suitable substrate for investigating the temperature responsiveness of genipin crosslinking because it has free amino residues and the solutions containing no genipin are stable at temperatures ranging from 25 to 37°C (data not shown).  Figure 9(a) shows the change in *G*′ of a 0.5% chitosan/5 mM genipin solution with increase in temperature from 25 to 37°C. *G*′ of the chitosan solutions began to increase approximately 60 s after the temperature reached 37°C. Only a slight increase in *G*′ was observed when the temperature was kept at 25°C (Figure 9(b)). These results indicated that genipin crosslinking was activated with increasing temperature from 25 to 37°C.

**Figure 10:**
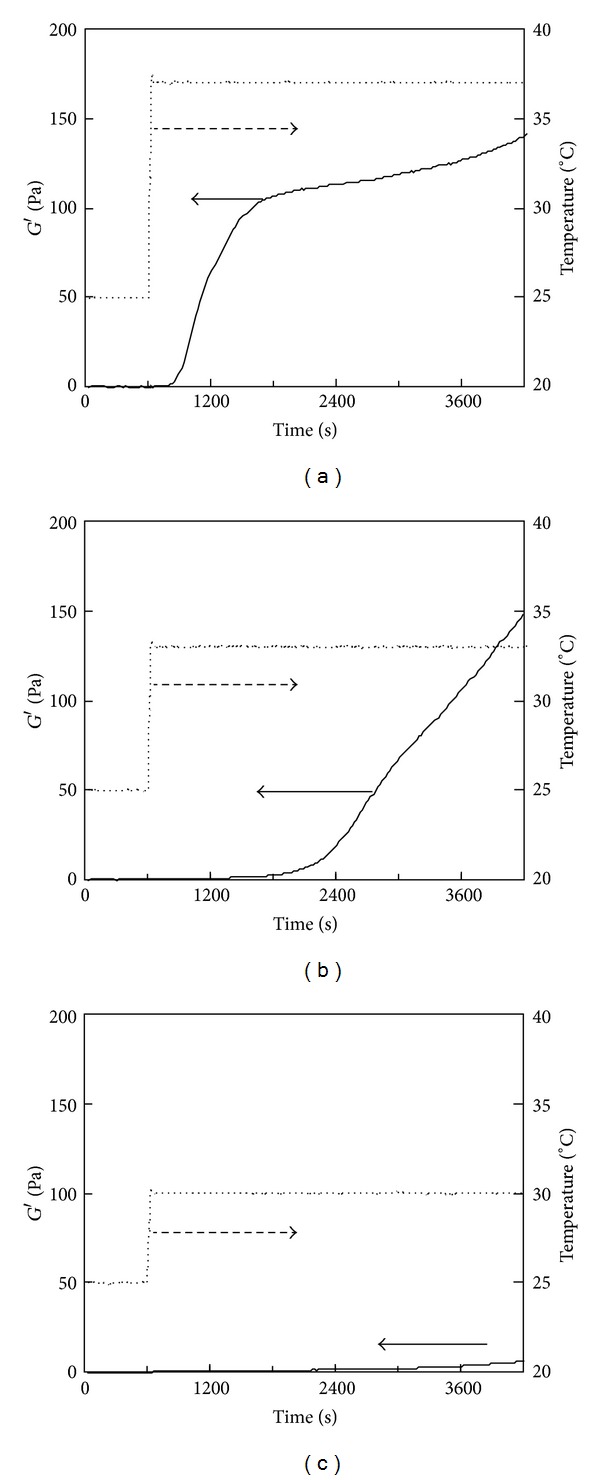
Storage modulus (*G*′) of the 0.5% PSC solution containing no genipin with increase in temperature. The temperature was maintained at 25°C for 600 s and then increased to (a) 37, (b) 33, or (c) 30°C.

The temperature dependence of collagen fibril formation without the influence of genipin crosslinking was also evaluated.  Figure 10 shows the change in *G*′ of a 0.5% PSC solution containing no genipin with increase in temperature from 25 to 30, 33, and 37°C. The initial rise in *G*′ decelerated with the target temperature. At the target temperature of 30°C, only a slight increase in *G*′ (6 Pa) was observed in 60 min. 

### 3.7. Comparison of Temperature Responsibility of GA and Genipin Crosslinking

The 0.5% PSC/6 mM GA was subjected to the fluidity test and temperature-responsive gelation test to compare temperature responsiveness between genipin and GA crosslinking.  Figure 9(a) shows the change in *G*′ of a 0.5% chitosan/6 mM GA solution compared with that of a 0.5% chitosan/5 mM genipin solution. Although GA concentration was higher than genipin concentration, the rate of increase in *G*′ of the chitosan/GA solution was lower than that of the chitosan/genipin solution. The fluidity test determined the gel points to be 780 ± 70 s (mean ± SD; *n* = 3) for the 0.5% PSC/6 mM GA solution and 1000 ± 120 s (mean ± SD; *n* = 3) for the 0.5% PSC/5 mM genipin solution, indicating that GA crosslinking was faster than genipin crosslinking at room temperature.

Representative gelation curves of both the solutions obtained by the temperature-responsive gelation test are shown in Figure 8. The 0.5% PSC/5 mM genipin solution showed a higher rate of increase in *G*′ than the 0.5% PSC/6 mM GA solution, whereas the fluidity test of chitosan showed that crosslinking of 5 mM genipin at 25°C was slower than that of 6 mM GA. The difference in the temperature-responsive gelation tests was confirmed by triplicate experiments. These results indicated that the temperature responsiveness of genipin crosslinking was higher than that of GA crosslinking.

### 3.8. Penetration Tests for Matured PSC/Genipin Gels

**Figure 11:**
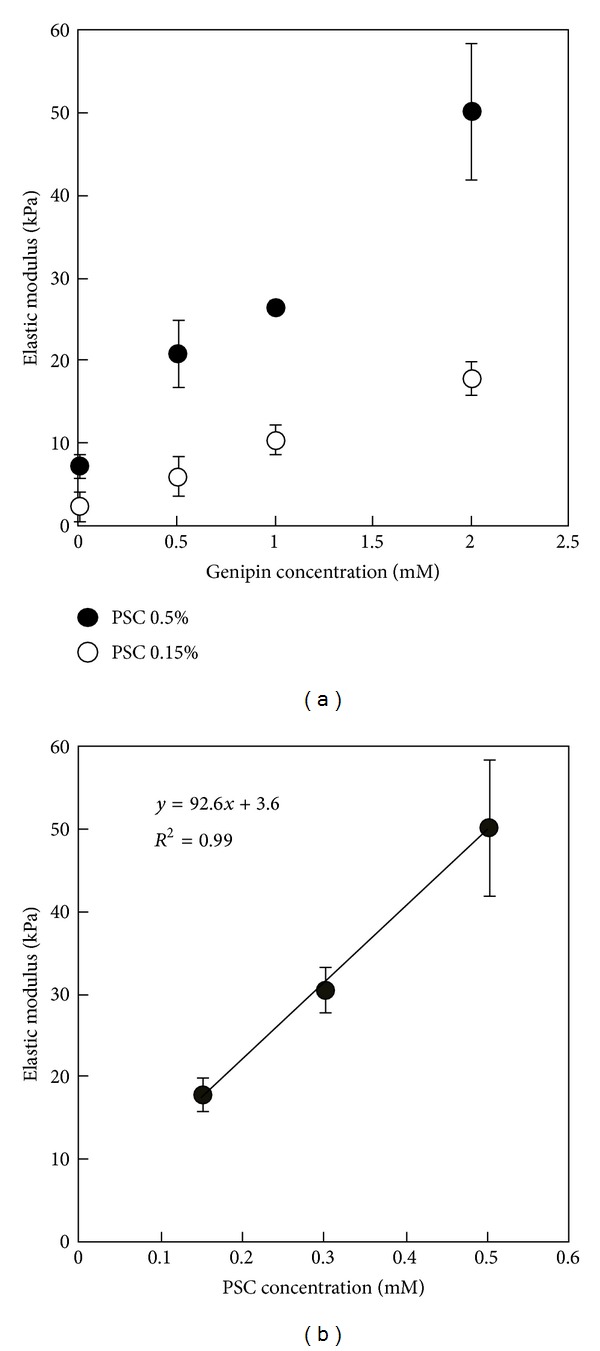
Elastic modulus of matured PSC/genipin gels obtained by a penetration test. (a) 0.5 and 0.15 wt% PSC gels containing genipin at concentrations ranging from 0 to 4 mM. (b) 0.5, 0.3, and 0.15 wt% PSC gels containing 2 mM genipin. The gels were matured by incubation for 72 h. Data are presented as mean ± SD; *n* = 3.

Figure 11 shows the elastic modulus of matured PSC/genipin gels obtained by the penetration test as a function of genipin and PSC concentrations. Plots of the elastic modulus against the genipin concentration are almost linear for both 0.5% and 0.15% PSC/genipin gels (Figure 11(a)). Only genipin concentrations higher than 0.5 mM markedly increased the stiffness of PSC gels when compared with the stiffness of gels containing no genipin. The elastic moduli of 0.5% and 0.15% PSC/genipin gels reached 50.2 ± 8.2 kPa and 17.9 ± 2.0 kPa, respectively, at a genipin concentration of 2 mM. The almost linear correlation between elastic modulus and PSC concentration at a constant genipin concentration of 2 mM yielded the following formula for the gels:

(1) $$\begin{array}{c} Y=92.6\cdot X+3.6, \end{array}$$

where *Y* is the elastic modulus (kPa) and *X* is the PSC concentration (%). Based on the linear correlation, a 0.1% increase in the PSC concentration corresponds to an approximately 9 kPa increase in the elastic modulus.

### 3.9. Time-Dependent Change of Activity of Genipin Crosslinking

**Figure 12:**
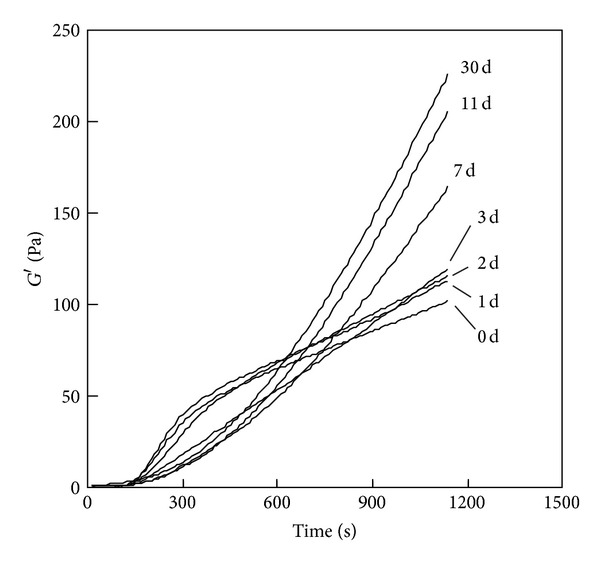
Storage modulus (*G*′) of the 0.25% PSC/10 mM genipin solution measured by a temperature-responsive gelation test. The numbers in the figure indicate the elapsed days after the preparation of genipin solution. The curves obtained at 37°C after the intervals of 600 s at 25°C were depicted.

To test time-dependent change of activity of genipin crosslinking, temperature-responsive gelation tests were conducted for the 0.25% PSC/10 mM genipin solutions by using genipin solutions prepared 0–30 d before the tests.  Figure 12 shows the curves obtained at 37°C after the intervals of 600 s at 25°C. The shapes of gelatin curves were similar for samples using genipin solution 0, 1, 2, and 3 d elapsed after the preparation. When the elapsed time increased from 3 to 7 d, the sigmoidal curves changed to J-like curves. The slope of the curves increased as the elapsed time increased and almost stabilized in the elapsed time of 11 d.

## 4. Discussion

 We demonstrated that temperature-responsive gelation occurred in PSC/genipin solutions at genipin concentrations 0–2 mM under a moderate change in temperature from 25 to 37°C, while the solutions exhibited fluidity at room temperature for at least 30 min. In specific cases this stability at room temperature could be an important property for injectable collagen, while fluidity of PSC/genipin solutions can be maintained at lower temperatures. In practical applications, there is a possibility that temperatures of injectable collagen increase to room temperature during operations depending on environments (especially on room temperatures), operation procedures, equipment, and skills of operators. The fluidity at 25°C therefore warrants easy handling of injectable collagen without unexpected gelation during various operations such as degassing the solutions or incorporating drugs and cells uniformly. The rapid gelation behavior under physiological conditions is beneficial not only for drug and cell entrapment, to yield uniform distributions within the gelled matrix, but also for creating required shapes and sizes in the body. A desirable gelation time appears to be within several minutes for minimizing diffusion of such contents, collagen, and genipin. The PSC/genipin gel system appears to satisfy the requirements for the physical properties of an injectable gel system.

 The temperature-responsive gelation of the PSC/genipin solutions was because of the temperature responsiveness of genipin crosslinking and collagen fibril formation. Investigations using the chitosan/genipin gel system revealed that genipin crosslinking itself was rapidly activated with increase in temperature from 25 to 37°C. Furthermore, PSC itself showed well-known gelation by fibril formation in response to increase in temperature from 25 to 37°C. The PSC/genipin gels consisted of collagen fibrils. From these findings, it was evident that not only collagen fibril formation but also genipin crosslinking contributed to the temperature responsiveness of gelation of the PSC/genipin solutions under such moderate changes in temperature.

The continuous increase in *G*′ of the PSC/genipin gels after temperature reached 37°C appeared to be predominantly due to genipin crosslinking, according to the findings that collagen fibril formation in the absence of genipin was almost stabilized in 15 min and that genipin crosslinking to chitosan occurred continuously for at least 60 min. Genipin crosslinking on collagen has been reported to continue for up to 3 days depending on the genipin concentration [25]. 

Special attention should be given for ASC/genipin system because of the rapid loss of fluidity at room temperature: the use of a refrigerator or an ice bath appropriately to maintain low temperature. The loss of fluidity and gelation was predominantly due to collagen fibril formation, in which genipin crosslinking would not substantially affect the early stage of the gelation according to our data for genipin crosslinking to chitosan at 25°C. Rapid fibril formation of ASC compared with that of PSC is thought to be because of the existence of nucleation centers derived from the oligomeric structure [32] and the presence of telopeptide regions important for stabilizing the collagen structure [28]. In Macaya's pioneer study of a collagen/genipin gel system [25], ASC from rat tail tendon was used, and rapid increase in *G*′ was observed approximately 50 s after the collagen/genipin solution was subjected to rheological tests. Those results were reproduced by our experiments using ASC from porcine tendon. We recommend that PSC be used for a collagen/genipin gel system to preserve fluidity at room temperature, offering sufficient time to degas the solutions and uniformly incorporate cells and drugs into the gels. 

 The temperature responsiveness of genipin crosslinking was greater than that of conventional GA crosslinking. Both GA and genipin crosslink free amino residues of polymer substrates, where the crosslinkers are oligomeric and polymeric forms [23, 24]. It appears that the genipin is a preferable crosslinker for temperature-responsive gelation of collagen with respect to temperature responsiveness of crosslinking as well as biological safety. 

The elastic modulus of the 0.5% PSC/genipin gel system can be adjusted over a range from 2.5 to 50 kPa by the PSC and genipin concentrations, and increases in the PSC concentration are expected to further increase the elastic modulus. The PSC/genipin gel system allows control of the elastic modulus of injectable collagen gels to match those of living tissues: brain tissue (approximately 0.3 kPa) [33], muscle (approximately 10 kPa) [34], and the collagen-based precursor to bone (approximately 30 kPa) [35]. Furthermore, the mechanical properties of the collagen gel contribute to maintain the shape of the gel in living tissues. However, direct cell exposure to genipin has been reported to pose serious cytotoxicity risks [24–26]. According to these reports using various cell types, the genipin concentration limit in injectable gels appears to be below 0.5–1.0 mM, except for neural stem cells [25]. Based on the above findings of cytotoxicity of genipin and the fact that wt% of collagen had a substantial effect on the modulus (Figure 8), the elastic modulus should be controlled by the collagen concentration rather than the genipin concentration. 

The elastic modulus determined by penetration tests may be different from that by dynamic viscoelastic tests because of differences in deformation modes and ranges. Penetration tests confer compressive deformation in the gels, while shear deformation is generated by dynamic viscoelastic tests. However, the shear modulus of the matured PSC/genipin gels could not be precisely measured by dynamic viscoelastic tests. We learned that it is difficult to determine shear modulus precisely when the matured gels prepared in containers are moved to a rheometer and subsequently measured. The difficulty is probably due to the leakage of water from the gels by continuous stress generated by a plate sensor. 

The limitation of PSC/genipin gel system appears to be a spontaneous crosslinking of collagen in PSC/genipin solutions that occurs even in a refrigerator, making it difficult to stock and distribute the mixed solutions. In practical uses PSC and genipin solutions should be stocked separately and mixed just before operations. We must note that activity of genipin crosslinking increases over a period of a week after genipin is dissolved in PBS. That is the reason that we used genipin solutions stocked in a refrigerator for over 2 weeks. A possible explanation for the activity change is self-polymerization of genipin in aqueous solution described by Sung et al. [21]. Such structural changes may alter the activity of intermolecular and intramolecular crosslinking or interference with collagen fibril formation. If dried genipin powder is dissolved in PBS just before operation, a time-course change in activity of genipin crosslinking should be investigated.

This study lacks biocompatibility data of PSC/genipin gels, which is also the limitation of this study. The biocompatibility of PSC has been well established. However, cytotoxicity of genipin seems to be a complex event because it is probably affected by mobility as well as activity of genipin. Our study demonstrated that activity of genipin is affected by temperature. Gelation of PSC/genipin solution would decrease the mobility of gelation, decreasing cytotoxic effects of genipin. The in vitro and in vivo biocompatibility data of PSC/genipin system are in progress to demonstrate the cytotoxicity of this injectable gel system. 

## 5. Conclusions

We demonstrated that PSC/genipin solutions are a potential injectable gel system: (1) fluidity was maintained at 25°C for at least 30 min, (2) a subsequent increase in temperature from 25 to 37°C caused rapid gelation, and (3) an elastic modulus markedly higher than that of conventional collagen fibrillar gels can be obtained at a genipin concentration of 0.5 mM, at which cells other than neural stem cells can survive direct contact with genipin. In specific cases PSC would be preferred over ASC as an injectable gel system. Temperature-responsive gelation of PSC/genipin solutions involves temperature responsiveness of both genipin crosslinking and collagen fibril formation. In vivo studies are now in progress to test mechanical properties and cytotoxicity of PSC/genipin gel system.
